# Reply to Comment on Detection of Mycotoxin in Patients with Chronic Fatigue Syndrome. *Toxins* 2013, *5*, 605-617 by Mark J. Mendell †

**DOI:** 10.3390/toxins8110325

**Published:** 2016-11-07

**Authors:** Joseph Brewer, Jack Dwayne Thrasher, Dennis Hooper

**Affiliations:** 1 Plaza Infectious Disease and St. Luke’s Hospital, 4320 Wornall Road, Suite 440, Kansas City, MO 64111, USA; 2 Citrus Heights, CA 95610, USA; toxicologist1@msn.com; 3 RealTime Laboratories, Carrollton, TX 75010, USA; dhooper@realtimelab.com

The authors of [1] have received further correspondence from Mark J. Mendell [2] concerning the above paper. We strongly disagree that the case series, which is reported by Brewer, et al., has flawed methodologies and is unsuitable for publication in a peer-reviewed journal. We also disagree that the control group selected was inappropriate and thus results invalidate comparison and findings.

Mendell emphasizes throughout his document that this is in essence a case-control study. This is simply not true. In reviewing his comments, we must emphasize that he is reviewing this paper as an epidemiologist and not as a M.D. As many, if not all, epidemiologists are aware, the purpose of epidemiology is to establish associations, which may be causative or may reveal clues to causation [3]. Wang and Attia (2010) stated: “to study causes or exposures known to be harmful, it is not ethical nor feasible to use an experimental design; for example, one cannot ask one group to start smoking and another to abstain from smoking to study if smoking causes age-related macular degeneration. Observational studies do not interfere in human subjects’ choice of exposure and assess outcomes in subjects who were exposed or not exposed to the factors of interest; these are surveys, case-control, cohort studies (all with controls) or case series (without controls)” [3]. Kempen, in 2011, stated the uncontrolled case series may suffer from a fundamental defect of lacking a contemporaneous comparison group which then leaves authors and readers to resort to historical controls [4]. He continues to state that observational case series make up a substantial proportion of publications submitted to journals (in his case, ophthalmic journals), which aspire to promulgate generalizable knowledge. When these studies are appropriately used, they serve an important and legitimate purpose in furthering medical knowledge, particularly when a question of importance cannot be addressed by other methods because of ethical or logistical constraints.

The Brewer paper reports a case series from a clinician who treats patients. Thus, reporting of a case series, such as the Brewer paper, adds to generalizable knowledge. Brewer et al. made no causal inferences from this case series.

Kempen states that observational case series receive very little attention among epidemiologists because of the limitations of no control [4]. This does not mean in any way that the observations reported are not meaningful and potentially helpful to care givers and their patients.

Kooistra et al. furthermore stated that case reports and case series that lack comparison groups might present data that is biased and incomplete [5]. Despite that, studies like this one are useful for generating hypotheses for future studies.

We understand the issues that Mendell cites but strongly disagree with his assessment. Mendell gives his points as an epidemiologist, the authors of Brewer, et al., point out the medical interpretation of such data and do not emphasize that this is an epidemiology study. To not publish these data or other case series would be limiting further future hypotheses and future studies in the area of chronic fatigue and mycotoxins.
